# Exploring User Learnability and Learning Performance in an App for Depression: Usability Study

**DOI:** 10.2196/humanfactors.7951

**Published:** 2017-08-11

**Authors:** Colleen Stiles-Shields, Enid Montague, Emily G Lattie, Stephen M Schueller, Mary J Kwasny, David C Mohr

**Affiliations:** ^1^ Center for Behavioral Intervention Technologies Department of Preventive Medicine Northwestern University Feinberg School of Medicine Chicago, IL United States; ^2^ Department of Psychiatry and Behavioral Neuroscience The University of Chicago Medicine Chicago, IL United States; ^3^ College of Computing DePaul University Chicago, IL United States

**Keywords:** apps, learning, cognitive therapy, usability testing, depression

## Abstract

**Background:**

Mental health apps tend to be narrow in their functioning, with their focus mostly being on tracking, management, or psychoeducation. It is unclear what capability such apps have to facilitate a change in users, particularly in terms of learning key constructs relating to behavioral interventions. Thought Challenger (CBITs, Chicago) is a skill-building app that engages users in cognitive restructuring, a core component of cognitive therapy (CT) for depression.

**Objective:**

The purpose of this study was to evaluate the learnability and learning performance of users following initial use of Thought Challenger.

**Methods:**

Twenty adults completed in-lab usability testing of Thought Challenger, which comprised two interactions with the app. Learnability was measured via completion times, error rates, and psychologist ratings of user entries in the app; learning performance was measured via a test of CT knowledge and skills. Nonparametric tests were conducted to evaluate the difference between individuals with no or mild depression to those with moderate to severe depression, as well as differences in completion times and pre- and posttests.

**Results:**

Across the two interactions, the majority of completion times were found to be acceptable (5 min or less), with minimal errors (1.2%, 10/840) and successful completion of CT thought records. Furthermore, CT knowledge and skills significantly improved after the initial use of Thought Challenger (*P*=.009).

**Conclusions:**

The learning objectives for Thought Challenger during initial uses were successfully met in an evaluation with likely end users. The findings therefore suggest that apps are capable of providing users with opportunities for learning of intervention skills.

## Introduction

### Mental Health Apps

Commercially available mental health apps have been rapidly emerging over recent years, and demand for them is high [1,2]. Roughly two-thirds of Americans own smartphones, and nearly 20% of all Americans rely on this technology as their only method for Internet access [3]. Additionally, 80% of Americans use the Internet for some form of digital health purposes, including searching for health information or tracking health-related factors [4]. This tremendous growth in smartphone ownership and the use of the Internet for health purposes has made it an attractive avenue for the delivery of behavioral health interventions via apps. Apps are accessible for independent download on app stores or may be used in conjunction with ongoing psychotherapy or with the support of a professional or paraprofessional [5-7].

Most apps with a focus on mental health are designed with a narrow functionality, focusing primarily on providing information to users as a way to enhance learning about their mental health symptoms or their management [5,8]. One categorization of their functionality used the following groupings: informing, instructing, recording, displaying, guiding, alerting, or communicating with users. Most apps fell into the grouping of informing (through the dissemination of psychoeducation), with a growing number of apps falling under the grouping of instructing [8]. Apps intended for instruction are skills-based, such that they enable the practice of specific intervention skills in a user’s own daily environment (ie, practicing a skill on a mobile device during daily life).

One such skills-based app is Thought Challenger, an app currently available through the Google Play Store [9]. Thought Challenger is one app in the IntelliCare suite, a collection of apps in which each app focuses on one behavioral strategy commonly used in the treatment of depression or anxiety [10,11]. Thought Challenger instructs users in the process of cognitive restructuring, the core strategy in cognitive therapy (CT) that involves identifying and appraising maladaptive thoughts and creating adaptive counter thoughts [12]. Thus, Thought Challenger is intended to teach users this specific CT skill and to help build mastery in this skill through repeated practice. Users are expected to use Thought Challenger on an as-needed basis and are prompted to return to the app through notifications. However, the interactions with Thought Challenger remain constant over time. It is therefore important to explore how effective Thought Challenger is, and how other instructive apps might be, at teaching this core skill.

### Learning in Cognitive Therapy as a Framework for Learning in Thought Challenger

The focus of CT is on educating patients about the impact of their thoughts on their mood while demonstrating how identifying, appraising, and modifying thoughts can lead to ultimate symptom reduction [12]. Patient learning and application of skills are noted to be among the possible mechanisms supporting symptom change in cognitive interventions [13,14]. Thought Challenger was designed to promote the learning and application of skills associated with symptom change in CT. However, the effectiveness of Thought Challenger in achieving this design aim is unknown.

The effectiveness of behavioral health intervention apps to achieve proximal goals purported to lead to ultimate symptom change is rarely evaluated. Apps are most often evaluated using randomized controlled trials; many researchers, however, have noted the limitations of these trials in the evaluation of mobile app technologies [15-17]. As such, it makes sense to leverage evaluation methodologies that are better suited for mobile technologies. One example would be usability testing, which is a method of evaluation that involves testing users’ interactions with a product and system to improve design. This process is intended to ensure that a technology is intuitive and easy to use. Usability testing requires systematic observation of a planned task or scenario carried out by an actual or potential user [18]. The International Organization for Standardization provides standards for usability testing, which define how to identify the information necessary for a designer to consider when specifying or evaluating usability of an evaluated product [19]. These techniques are used in engineering and computer science to evaluate and refine products, and are being used with increasing frequency in the context of behavioral health interventions delivered via technologies [20-22]. Indeed, usability testing is an ideal methodology to systematically examine users’ learning of CT skills because of interactions with a mobile behavioral health intervention, such as Thought Challenger.

It is also important to evaluate how well a user will learn a depression intervention skill through the use of an app, without first reviewing any instructions. The evaluation of learning without instruction is important, given that most users are unlikely to engage with instructions or help materials before use, despite the likely benefits of doing so [23]. This behavior is referred to as the Paradox of the Active User and has been found to extend to the use of apps [24]; it helps to explain why users may be quick to reject apps that are initially perceived as not meeting their needs, even when detailed “Help” or “FAQ” sections exist. Therefore, apps should be able to achieve their aims through intuitive design [25]. Thus, evaluating the first-time user experience of an app such as Thought Challenger is critical, as this initial experience shapes subsequent use (or nonuse).

### Purpose

Despite the growth in skills-based apps for mental health, the efficacy of such apps in promoting skills-based learning through their use is unknown. Furthermore, it has recently been documented that mental health providers may have concerns about the credibility and risk associated with treatment provided via mobile phone apps [6,26] and may be skeptical about the capabilities of such apps. The purpose of this study is to understand CT skill learning in the context of an app for depression, Thought Challenger, via usability testing methodologies. This study tested three learning objectives to evaluate the efficacy of the app, which included: (1) how well a user initially interacts with the Thought Challenger app without instruction; (2) the user’s ability to learn the skill of cognitive restructuring from the app; and (3) the effect of using Thought Challenger on knowledge of CT elements.

## Methods

We will first describe Thought Challenger, following the framework for the evaluation of the app, and the specific procedures of the usability testing.

### Thought Challenger

Thought Challenger, currently available through the Google Play Store, was informed by CT. It was specifically designed to aid users in engaging in the CT-based technique of thought restructuring. This process involves identifying thought distortions, which are unhelpful or erroneous thoughts that occur automatically but cause distress or mood changes in a person. Following the identification of such thought distortions, thought restructuring involves asking oneself questions to help challenge this distorted thought and to come up with a more helpful alternative thought [12].

Thought Challenger has two functions: challenge and review. The challenge feature is a tool designed to help restructure each thought through 5 steps: (1) “Catch It”: enter a recent maladaptive thought; (2) “Check It”: reflective questions are posed regarding the thought; (3) “Choose a Distortion”: identify in which type of cognitive distortion the thought likely falls; (4) Consider reflective questions tailored to the chosen type of distortion; and (5) “Change It”: enter a more adaptive thought. Within steps 1 and 5, Thought Challenger provides examples of possible maladaptive and adaptive thoughts, which users may select and use in their interaction with the thought restructuring tool. Thought Challenger also provides a review function so that users can see their past entries of all thoughts, listed by automatic thought, rational response, distortion, and date and time of interaction.

### Framework

Attributes are usability features that measure different usability qualities of technology products [27]. Table 1 displays the usability attributes, learnability, and learning performance used to measure the learning of users with Thought Challenger. The tasks, measurement, and objectives used in this evaluation are detailed below.

**Table 1:** Usability attributes and their application to learning evaluation.

Qualifier	Learnability	Learning performance
Description	Level of ease through which a user gains proficiency	Actual impact on performance of a task/acquisition of knowledge
Tasks for testing	Complete two attempts at using the Thought Challenger tool	Complete a pre-and posttest of cognitive therapy and skills
Measurement via	Time to complete interactions Error rate Rating of completed thought record	Scores on pre-and posttest
Learning objectives	Identify how user interacts without instruction or didactic material Examine whether user learns to use the app within an acceptable time limit, with a low error rate	Measure change in knowledge of cognitive therapy skills and concepts following initial use

#### Learnability

Learnability is defined as the level of ease through which a user gains proficiency with an app [28]. Learnability of the Thought Challenger tool was ascertained through multiple methods. First, *time to completion* for unguided interactions with the tool was measured across two separate attempts. As users report spending about 5 min or less to learn how to use an app [29], successful time to completion was defined as an interaction completion time of 5 min or less. Second, learnability was measured by *error rate*. Errors were categorized as slips (ie, an unintended action with the correct goal, such as a typo), mistakes (ie, a behavior with an incorrect goal, such as typing in today’s date rather than a date of birth), or fatal errors (ie, an error that prevents the user from completing the task even with provided instruction/guidance) [30,31]. Error rates were obtained by dividing the total number of errors made by the number of error opportunities. Error opportunities are the total number of actions a user must complete to finish an interaction without errors [32]. For the purposes of the structured interaction with Thought Challenger, the number of error opportunities was 21. To the best of our knowledge, the literature does not define an ideal error rate for initial app use. Therefore, error rate was established for this app, along with the identification of any violated usability heuristics (ie, general principles of design). Third, learnability will also be measured via the *number of accurately completed thought* *records* using the Thought Challenger app. Thought restructuring can be a difficult skill for patients to grasp on initial attempts [12,33,34]. A successful rate for this measure of learnability will be that licensed psychologists experienced in administering thought records in the course of CT will rate 63% or more of entries into the app as accurately completed for the skill of thought restructuring. This rate is based upon the findings of patient abilities to accurately complete thought records on their own during face-to-face delivery of cognitive interventions [33].

#### Learning Performance

Learning performance is an attribute of usability relating to the actual impact of a technology on the performance of a task or acquisition of knowledge, such as the ability of a technology to aid in increasing capabilities to complete assignments in a classroom [35]. As the testing of this study occurred during single in-lab sessions, learning performance was measured via *scores on a pre/posttest* of CT knowledge and skills. Successful learning performance was defined in this study as a significant increase in the score of a questionnaire evaluating CT knowledge and skills in a pre/posttest administration. Learning performance was measured in this testing as a means of evaluating objective 3, that is, measure change in the knowledge of CT intervention elements following initial use of Thought Challenger.

### Recruitment

Recruitment of participants occurred from July to August 2015 from Web-based postings in the Chicago area of the United States, resulting in the participation of 20 adults. Inclusion criteria required that participants were at least 18 years of age, able to attend an in-lab testing session, and able to speak and read in English. As depression is a condition that is frequently chronic, characterized by patterns of remissions and relapses [36-38], equal numbers of participants currently above and below the criteria for a referral for psychotherapy were recruited [39]. This sampling ensured that learning objectives were being measured with likely end users, ranging from those with no or mild depressive symptoms (subthreshold for a referral to psychotherapy as measured by a Patient Health Questionnaire-9 [PHQ-9] score of less than 10) to those with moderate or severe depressive symptoms (threshold for a referral to psychotherapy as measured by a PHQ-9 score greater than or equal to 10) [40]. Participants who completed in-lab usability sessions were compensated US $20 in petty cash for their time and participation. In compliance with the University’s institutional review board (IRB), participants completed a Web-based screening consent before the collection of any data and were consented in person for the usability testing session.

### Procedure

Participants were invited to a laboratory room located within Northwestern University’s Feinberg School of Medicine and were accompanied by a moderator, who provided guidance and noted participants’ actions throughout the testing session. Before the testing of Thought Challenger, participants engaged in a card-sorting task to identify the barriers to the use of apps for depression [41]. Following this, participants were provided a description of the app, which is also listed in the Google Play Store site when one would download the app: “Thought Challenger helps you gain control of how you feel and what you do by teaching you to notice and challenge negative and unhelpful thoughts. Thought Challenger is built on cognitive therapy - a structure that has been found in clinical studies to be useful in examining negative thoughts and reframing them to help you feel better and do the things you want to do” [9]. Users were then instructed to pick up the Android phone used for testing (lying on table directly in front of user), open the Thought Challenger app, challenge a recent negative thought, and inform the testing moderator when the user believed the task was completed. The interaction was timed and recorded, and the moderator wrote down any observed errors and alternative paths made in completing the first interaction. Users were then queried about any alternative paths taken to complete the interaction, whether they were able to find the log of the tool interaction they just completed, and whether they were able to find more information about the app (ie, Frequently Asked Questions or Help sections). These interactions were also recorded and timed and allowed for a delay between the two challenge tool interactions measured. Once completed, the users were prompted: “Now, please log another recent negative or unhelpful thought you have had.” This interaction was also timed and observed, and all entries into the tool were recorded for later review. Participants therefore had two complete interactions with the Thought Challenger tool during the evaluation. Following a brief interview of the user impressions of Thought Challenger, users completed questionnaires on a lab computer.

### Data Collection Approaches

Traditional data collection methodologies, which have been successfully used in other evaluations of apps [21,28,42], were chosen for the testing of Thought Challenger. Specifically, data collection included the following: (1) video/audio recording of the interactions; (2) standardized interview questions with the option to prompt regarding specific behaviors or observations; (3) questionnaires (see Measures section); (4) timing of all interactions via stop watch; and (5) recording of all user actions into the app’s thought restructuring tool (ie, entry of thought and assignment of type of thought distortion).

### Measures

Study data were collected and managed using REDCap (Research Electronic Data Capture) tools hosted at Northwestern University [43]. REDCap is a secure, Web-based application designed to support data capture for research studies, providing the following: (1) an intuitive interface for validated data entry; (2) audit trails for tracking data manipulation and export procedures; (3) automated export procedures for seamless data downloads to common statistical packages; and (4) procedures for importing data from external sources.

At screening, the participants were asked to provide demographic information (ie, gender, race/ethnicity, age, education, and employment status). Thereafter, they completed the PHQ-9 and CT Tool Knowledge and Skill Pretest at screening [40,44]. Following the completion of the interactions with Thought Challenger in the usability testing session, participants completed the CT Tool Knowledge and Skill Posttest, which is identical to the Pretest.

The PHQ-9 is a 9-item self-report instrument measuring depressive symptomology with scores ranging from 0 to 27 [40]. The CT Tool Knowledge and Skill Pre/Posttest is a measure adapted from the Cognitive Therapy Awareness Scale (CTAS) [44]. The CTAS is a measure evaluating understanding of CT constructs and skills. The language in the CTAS was modified to reflect only language and concepts presented in the Thought Challenger app. The range of possible scores is 0 to 40. The CT Tool Knowledge and Skill Pre/Posttest were administered at screening (pre) and after interacting with the app during the testing session (post). These time points allowed for about 1 week’s delay between the pre- and posttest administration, with the intent of negating possible priming effects associated with pre/posttests.

### Data Analysis

The thought record entries in Thought Challenger were collected to measure success of users in Thought Challenger tool use, that is, identifying how accurately users engaged in thought restructuring on the app. Following the completion of all testing sessions, doctoral-level clinical psychologists blindly rated participants’ entries of maladaptive thoughts, assignment of type of cognitive distortion, and entries of alternative thoughts across their two interactions with the tool (such that each complete entry was rated by 2 separate psychologists). The psychologists were instructed to evaluate the entries as if they were thought records, a tool typically administered via paper, handed out in face-to-face CT to enable the practice of thought restructuring [12]. The ratings were binary, such that the psychologists rated each entry section as either accurately or inaccurately completed. When there was conflict in the psychologist ratings (each entry was rated by 2 psychologists), a third clinician was invited to provide consensus on the entry.

Given the small sample size and anticipated non-normal distribution (ie, participants ranging from no depressive symptoms to severe), nonparametric tests were conducted to analyze quantitative usability testing data. Wilcoxon signed-rank tests were used to analyze comparison of time to completion of the tool interaction on the first and second attempt, as well as comparison of scores before and after the interaction with Thought Challenger. To ensure that there were no significant differences between the participants recruited with PHQ-9 scores above and below 10, Mann-Whitney *U*-tests were performed to compare the participants on times to completion, total scores on completed measures, and demographic variables. Chi-square tests were completed to compare categorical demographic variables. All analyses were run in Statistical Package for the Social Sciences version 23 (IBM Corp), at the nominal 0.05 type I error rate.

## Results

### Participants

Table 2 displays the sample characteristics for the evaluation of Thought Challenger. One extra participant was recruited to the PHQ-9< 10 group, making the groups roughly equal. There was no significant difference between participants above and below the criteria for a referral for psychotherapy for age, gender, or race. Those meeting the criteria for a referral to psychotherapy had significantly higher depressive symptom severity (14.4 vs 3.8, *P*<.001) and a significantly higher prevalence of past depressive episodes (77.8% vs 18.2%, *P*=.008).

**Table 2:** Usability testing sample characteristics.

Demographic	PHQ-9^a^<10 (n=11)	PHQ-9≥10 (n=9)	Total (n=20)
Female, n (%)	7 (63.6)	8 (88.9)	15 (75)
Age in years, mean (standard deviation)	34.5 (10.3)	40.6 (14.0)	37.2 (12.2)
African American, n (%)	4 (36.4)	1 (16.7)	5 (25)
Asian, n (%)	2 (18.1)	0 (0)	2 (10)
Hispanic white, n (%)	1 (9.1)	0 (0)	1 (5)
Non-Hispanic white, n (%)	5 (45.5)	8 (88.9)	13 (65)
PHQ-9, mean (standard deviation)	3.8 (3.2)	14.4 (5.8)	8.6 (7.0)
History of depression, n (%)	2 (18.2)	7 (77.8)	9 (45)
History of anxiety, n (%)	2 (18.2)	5 (55.6)	7 (35)

^a^PHQ-9: Patient Health Questionnaire-9.

### Learnability

#### Completion Time

Table 3 displays the completion times for the Thought Challenger tool interactions. For all participants, the median time to complete an initial, unguided interaction with the Thought Challenger tool was 4:05 min. Sixty-five percent of the sample met the criterion requiring about 5 min or less for the first interaction [29]. Median time to complete the task on second attempt was significantly faster (4:05 vs 2:34, *P*=.001). Of note, the median times to complete the task across time points were identical for the PHQ-9≥10 group. However, the interquartile range (IQR) was smaller (7:30 vs 3:40), indicating that there was less variance in times on the second attempt for this group.

**Table 3:** Tool interaction completion times, median (interquartile range).

Time point	PHQ-9^a^<10	PHQ-9≥10	Total
Time 1	4:13 (4:01)	3:57 (7:30)	4:05 (4:04)
Time 2	2:08 (1:11)	3:57 (3:40)	2:34 (2:00)

^a^PHQ-9: Patient Health Questionnaire-9.

#### Error Rate

Ten errors occurred across the two interactions for each participant with the Thought Challenger tool. On the first attempt at the Thought Challenger challenge interaction, 9 mistakes were made, relating to attempts to interact with the Thought Challenger word cloud on the home screen (ie, clicking on the word cloud rather than a button), selecting “Review” rather than “Challenge” to begin to challenge a thought, and persistence in the remaining challenge interactions after first entering a maladaptive thought (eg, “I entered my thought in like it said, now what?”). No slips or fatal errors occurred for any participants across the first interaction.

On the second interaction with the Thought Challenger challenge tool, one fatal error occurred, preventing the user from completing the task even with provided instruction and guidance because of frustration saturation (ie, “I don’t want to start all over again and re-enter everything.”). This fatal error occurred by the user clicking “cancel” while entering data into the challenge tool. Thought Challenger brought the user back to the Thought Challenger home screen without saving the entered data and without prompting the user that data would be lost. This is an example of violating the usability heuristic of error prevention, which guides designers to reduce or eliminate conditions that are likely to lead to errors in interactions [27]. Of note, no slips occurred during the second interactions. Although participants had in-the-moment slips, such as typos, these were not maintained in the system because of the Android operating system’s algorithm to correct slips such as auto-populating words when a suspected typo occurs during text entry.

The total error rate for all initial interactions with the Thought Challenger tool was therefore defined by 10 (errors)/(21 [error opportunities] x 2 [number of interactions] x 20 [participants])=.012. Therefore, the error rate on initial interactions with Thought Challenger’s tool was 1.2%.

#### Successful Completion of Tool Records

The majority of tool entries were rated as appropriate by doctoral level psychologists, with 75% (30/40) success in entries of a maladaptive thought, 51% (20/39) success in choice of type of thought distortion, and 74% (29/39) success in the entry of an adaptive thought. Consistent with face-to-face findings, the rate of success was determined to be 63% or greater [33]. The ratings provided by doctoral-level clinical psychologists indicate learnability consistent with testing aims via the Thought Challenger tool.

### Learning Performance

#### Acquisition of Skills and Knowledge

To identify learning performance of users following use of Thought Challenger, all participants completed a pre- and posttest of CT skills and knowledge. Table 4 displays the medians and IQRs of pre- and posttest scores. A Wilcoxon signed-rank test indicated significant improvement in median scores for the entire sample, following the use of Thought Challenger (28.5 vs 31.0, *P*=.009). Successful learning performance was achieved for Thought Challenger, as there was a significant increase in performance on a CT knowledge and skills questionnaire following interactions with the app.

**Table 4:** Cognitive therapy pre-and posttest scores, median (interquartile range).

Time point	PHQ-9^a^<10	PHQ-9≥10	Total
Pretest	26.0 (11.0)	29.0 (5.5)	28.5 (11.3)
Posttest	29.0 (6.0)	32.0 (10.0)	31.0 (6.8)

^a^PHQ-9: Patient Health Questionnaire-9.

#### Consistent Performance Across Symptom Severity

No significant differences in completion times or in the performance on the pre- and posttest of CT skills and knowledge before or after interactions with Thought Challenger were identified between the two groups above and below the threshold for a referral to psychotherapy (*PS>*.13).

## Discussion

This study aimed to evaluate CT learning during initial interactions with a publicly deployed, skills-based app for depression [10,11]. Thought Challenger presents a challenge tool for thought restructuring without separate didactic material; it is learnable within an acceptable time frame for initial use of an app [29] and produces a low error rate. Results also indicate that the Thought Challenger tool promotes effective execution of thought restructuring and that CT knowledge and skills improve significantly after initial use. Ultimately, users are able to meet the learning objectives for Thought Challenger during initial use, indicating that skills-based apps can teach an intervention skill for depression through very brief interactions.

### Thought Challenger Performance

Thought Challenger met the evaluated learning objectives, creating entries in the tool that met the standard of accurately reflecting CT thought records at a rate of about 75%. This exceeded the benchmark of 63% of patients who were able to accurately complete the records as between-session homework throughout treatment [33]. One possible reason for the comparable performance of participants without the guidance of a therapist was that Thought Challenger provides the option of viewing example maladaptive and adaptive thoughts. However, in the 40 tool interactions in this testing, only 7 interactions (approximately 17%) employed example thoughts in the entries. Although not used frequently, the example thoughts may have provided a scaffold for participants to appropriately select and enter their own maladaptive and adaptive thoughts. Initial Thought Challenger entries are comparable in accuracy to thought records completed in the course of face-to-face interventions.

Thought Challenger was able to impact learning without requiring users to read or engage with didactic content. This is in contrast to most currently available mental health apps, which focus on providing information about symptoms and/or their management (ie, inform) [8]. Furthermore, when psychoeducation is presented in depression apps, a static interface is predominantly used (ie, similar to reading an e-book) [5]. Thought Challenger differs from this design by training users in a skill via interactive engagement with its tool. With continued use of the tool, users practice the skill of thought restructuring. Thought Challenger produced CT skills, demonstrated both through the ability to produce accurate thought records and by the significant improvement in user knowledge of the intended construct. This finding supports the idea that people can learn psychological constructs and skills solely through skills training apps, without psychoeducation.

### Opportunities for Improvement

Although Thought Challenger met the criteria for learnability and learning performance established for this study, the evaluation indicated opportunities for improvement of the app. First, a fatal error occurred (ie, an error that prevented the user from completing the task even with provided instruction/guidance) [30,31] in one user’s interaction with the app. This error violated the usability heuristic of error prevention [27], as this error could have been prevented through the use of a warning notification with the following options: (1) to warn the user that his/her data would not be saved if s/he continues with the action; or (2) offering the option to save the data for a later interaction before exiting to the home screen. Second, mistakes that occurred could likely be minimized through the usability heuristic of help and documentation [27]. In providing more guidance to users who might be confused by the options (ie, word cloud on home screen, whether to select “Review” or “Challenge” buttons), the likelihood of mistakes could be reduced. Evaluations of apps through RCTs are likely to miss such fatal errors, focusing instead on exploring whether the app generally leads to a clinical benefit for participants. The possibility for such errors within an app may be one reason that behavioral health apps show low rates of retention when deployed in public marketplaces [45]. As such, it is critical to explore the use of these resources through methodologies such as usability testing in addition to evaluating their efficacy through other methodologies.

### Limitations

There are several limitations and caveats that should be considered in interpreting these results. First, this was an evaluation of learnability and learning performance of Thought Challenger following initial use. It is unclear how these results would apply to long-term use, knowledge, skill application, or symptom reduction. Furthermore, as an evaluation of learning, there was no opportunity for comparison to other apps that promote learning (eg, different skills and psychoeducation only). Second, this study examined Thought Challenger in the context of users with symptom severity ranging from absent to severe depression, with the majority in the mild depressive range. It is unclear how these findings extend to users with other psychiatric or medical comorbidities. Third, while in-lab sessions were chosen over field-testing for multiple reasons, it is possible that the presence of a session moderator impacted user confidence or performance in a way that might have differed from field use. Finally, because of geographical limitations, the sample comprised urban and primarily younger users; it is unclear how well these findings extend to users in differing geographical locations and demographic groups.

### Future Direction

This study employed usability methodology [27], borrowed from the field of engineering, to provide insight into user learning from initial interactions with an app targeting users with depression. This was ultimately to promote the design and dissemination of treatment apps that can be both trusted by providers, and useful and usable for patients. There is a need for future research evaluating how skills-based learning and practice through apps impacts long-term symptom management. This work should also extend to other chronic conditions beyond depression, as currently available apps may not be consistently usable for diverse and vulnerable populations [46].

### Conclusions

To the best of our knowledge, this is the first use of usability testing methods to evaluate learning in an app intended to help users to learn and practice an intervention skill. Future research is needed to explore the role of learning in such apps and how to continue to improve skills-based learning, particularly in users with depression. This will promote improved design and dissemination of such apps. There has been some noted skepticism of clinicians on the efficacy of mental health apps. However, the findings from this study suggest that users can learn to complete a therapeutic intervention skill effectively through the use of a mobile tool alone, without engaging in didactic content.

## Abbreviations

CBITs: Center for Behavioral Intervention Technologies
CT: cognitive therapy
CTAS: Cognitive Therapy Awareness Scale
IQR: interquartile range
IRB: institutional review board
PHQ-9: Patient Health Questionnaire-9
REDCap: Research Electronic Data Capture
